# Supplementary material for: Sulfation affects apical extracellular matrix organization during development of the Drosophila embryonic salivary gland tube
Source: eLife. 2025 Sep 23;14:RP108292. doi: 10.7554/eLife.108292 (PMC12456955; doi:10.7554/eLife.108292)
Supplement: Supplementary file 1. [file elife-108292-supp1.docx]

**Supplementary File 1. A targeted Df screen revealed Papss as a key enzyme for SG lumen expansion.**

| **SG-upregulated enzymes** | **Predicted roles** | **Df lines tested** | **SG lumen shape** |
| --- | --- | --- | --- |
| CG17633 | Metallocarboxypeptidase | Exel6024 | Disrupted embryo morphology |
| CG30283 | Serine-type endopeptidase | Exel6076 | Disrupted embryo morphology |
| Papss | PAPS synthetase | Bsc445 | Thin SG lumen with apical protrusions |
| PH4αSG1/SG2 | Prolyl 4-hydroxylase | Exel6216 | Non-uniform lumen diameter |
| CG16758 | Purine nucleotide phosphorylase | Bsc116 | Non-uniform lumen diameter |
| CG8708 | N-acetylgalactosaminide β-1,3-galactosyltransferase | Exel7095 | Irregular apical membrane |
| CG7920 | Acetate CoA-transferase | Bsc620 | Irregular apical membrane |
| Pgant6 | Polypeptide N-acetylgalactosaminyltransferase | Exel6091 | Irregular apical membrane |
| GlcAT-P | Glucuronosyltransferase | Bsc817 | Irregular apical membrane |
| CG34290 | Serine-type endopeptidase | Bsc638 | Irregular apical membrane |
| Peroxidasin | Bromide peroxidase | Bsc119 | Irregular apical membrane |
| ScsβG | Succinyl Co-A synthetase β subunit | Bsc387 | Short and rounded SG |
