# Supplementary material for: Sulfation affects apical extracellular matrix organization during development of the Drosophila embryonic salivary gland tube
Source: eLife. 2025 Sep 23;14:RP108292. doi: 10.7554/eLife.108292 (PMC12456955; doi:10.7554/eLife.108292)
Supplement: Supplementary file 2. [file elife-108292-supp2.docx]

**Supplementary File 2. Fly strains used**

| **Fly strain** | **Sources and References** | **RRID** |
| --- | --- | --- |
| *Oregon R* (wild type) |  |  |
| *sage-Gal4* | Chung et al., 2009 |  |
| *fkh-Gal4* | Henderson and Andrew, 2000 |  |
| *Papss^2^* | Bloomington Stock Center | BDSC_26171 |
| *UAS-p35* | Bloomington Stock Center | BDSC_5072 |
| *UAS-Papss-PD* | This work |  |
| *UAS-Papss-PE* | This work |  |
| *UAS-Papss-PD^K193A, F593F^* | This work |  |
| *Dpy-YFP* | Kyoto stock center | DGGR_115238 |
| *SrpHemo3x-mCherry* | Gyoergy et al., 2018 | BDSC_78358 |
| *Dp-YFP, mCh-Qsm* | Chu and Hayashi, 2021 |  |
| *mCh-Qsm* | Chu and Hayashi, 2021 |  |
| *Dpy-YFP, mCh-Pio* | Drees et al., 2023 |  |
| *UAS-ManII-GFP* | Bloomington stock center | BDSC_65248 |
| *UAS-GFP-KDEL* | Bloomington stock center | BDSC_9898 |
| *pio^17c^* | Drees et al., 2023 |  |
| *Np^C2^* | Drees et al., 2023 |  |
| *dpy^ov1^* | Bloomington stock center | BDSC_276 |
| *Grasp65^102^* | Zhou et al., 2014 | BDSC_65257 |
| *GM130^Δ23^* | Zhou et al., 2014 | BDSC_65255 |
| *UAS-Np.WT-Strep* | Drees et al., 2019 |  |
| *UAS-Np.S990A-GFP* | Drees et al., 2019 |  |
