# Supplementary material for: Sulfation affects apical extracellular matrix organization during development of the Drosophila embryonic salivary gland tube
Source: eLife. 2025 Sep 23;14:RP108292. doi: 10.7554/eLife.108292 (PMC12456955; doi:10.7554/eLife.108292)
Supplement: Supplementary file 3. [file elife-108292-supp3.docx]

**Supplementary File 3. Antibodies Used**

| **Antibody** | **Source** | **RRID** | **Dilution** |
| --- | --- | --- | --- |
| α-Ecad (rat) | DSHB (DCAD2) | AB_528120 | 1:50 |
| α-CrebA (rat) | Andrew lab (John Hopkins University); re-made in this study |  | 1:3,000 |
| α-CrebA (rabbit) | Andrew lab (John Hopkins University) | AB_10805295 | 1:5,000 |
| α-CrebA (guinea pig) | This study |  | 1:5,000 |
| α-GFP (mouse) | Invitrogen (A11120) | AB_221568 | 1:500 |
| α-GFP (chicken) | Invitrogen (A10262) | AB_2534023 | 1:1,000 |
| α-RFP (rabbit) | Invitrogen (R10367) | AB_10563941 | 1: 2,000 |
| α-β-galactosidase (rabbit) | Invitrogen (A11132) | AB_221539 | 1:500 |
| α-β-galactosidase (mouse) | Invitrogen (MA5-15222) | AB_10980770 | 1:500 |
| α-mCherry (rat) | Invitrogen (M11217) | AB_2536611 | 1:1,000 |
| α-mCherry (rabbit) | Invitrogen (PA534974) | AB_2552323 | 1:1,000 |
| α-DCP-1 (rabbit) | Cell signaling technology (9578) | AB_2721060 | 1:200 |
| α-Crb (mouse) | DSHB (Cq4) | AB_528181 | 1:10 |
| α-Rab11 (rabbit) | Andrew lab (John Hopkins University) |  | 1:500 |
| α-Rab7 (mouse) | DSHB (Rab7) | AB_2722471 | 1:10 |
| α-Sec15 (guinea pig) | Bellen lab (Baylor College of Medicine) |  | 1:2,000 |
| α-α-Spectrin (mouse) | DSHB (3A9) | AB_528473 | 1:2 |
| α-Discs large (mouse) | DSHB (4F3) | AB_528203 | 1:500 |
| α-Pio (rabbit) | Affolter lab (Jaźwińska et al., 2003) |  | 1:500 |
| α-Nidogen (rabbit) | Holz lab (Wolfstetter et al., 2009) |  | 1:2,000 |
| α-Sulfotyrosine (mouse) | Sigma (ZMS1096-25UL) |  | 1:50 |
| Alexa Fluor 488/568/647- coupled secondary antibodies | Invitrogen |  | 1:500 |
